# Clinical Outcomes of Ascending Aortic Replacement for Aneurysm and the Impact of Centre Volume: A Nationwide Population-Based Study

**DOI:** 10.1093/icvts/ivag184

**Published:** 2026-07-27

**Authors:** Sue Hyun Kim, Jinhee Kim, Yoonjin Kang, Suk Ho Sohn, Yeiwon Lee, Ho Young Hwang, Kyung Hwan Kim, Mi-Sook Kim, Jae Woong Choi

**Affiliations:** Department of Critical Care, Seoul National University Hospital, Seoul 03080, Republic of Korea; Division of Clinical Epidemiology, Medical Research Collaborating Center, Biomedical Research Institute, Seoul National University Hospital, Seoul 03080, Republic of Korea; Department of Thoracic and Cardiovascular Surgery, Seoul National University Hospital, Seoul National University College of Medicine, Seoul 03080, Republic of Korea; Department of Thoracic and Cardiovascular Surgery, Seoul National University Hospital, Seoul National University College of Medicine, Seoul 03080, Republic of Korea; Department of Critical Care, Seoul National University Hospital, Seoul 03080, Republic of Korea; Department of Thoracic and Cardiovascular Surgery, Seoul National University Hospital, Seoul National University College of Medicine, Seoul 03080, Republic of Korea; Department of Thoracic and Cardiovascular Surgery, Seoul National University Hospital, Seoul National University College of Medicine, Seoul 03080, Republic of Korea; Division of Clinical Epidemiology, Medical Research Collaborating Center, Biomedical Research Institute, Seoul National University Hospital, Seoul 03080, Republic of Korea; Department of Thoracic and Cardiovascular Surgery, Seoul National University Hospital, Seoul National University College of Medicine, Seoul 03080, Republic of Korea

**Keywords:** ascending aortic replacement, thoracic aortic aneurysm, volume-outcome. Relationship

## Abstract

**Objectives:**

To evaluate contemporary outcomes of elective ascending aortic replacement (AAR) and the impact of centre volume using a nationwide database.

**Methods:**

Adults undergoing elective AAR for aneurysms (2003-2021) were identified from the Korean National Health Insurance Service database. Acute dissections, ruptures, and arch involvements were excluded. Restricted cubic splines (RCS) and multivariable regression evaluated the impact of institutional volume and concomitant procedures.

**Results:**

Among 5209 patients, operative mortality and stroke rates were 2.9% and 2.2%. Over a 5.9-year median follow-up, RCS revealed a significant non-linear inverse relationship between annual institutional volume and mortality. Treatment at high-volume centres (≥10 cases/year) independently lowered operative mortality (odds ratio, 0.26; 95% CI, 0.18-0.37) and long-term mortality (hazard ratio, 0.64; 95% CI, 0.57-0.73). Distinct clinical phenotypes, including bicuspid aortic valves and concomitant aortic valve surgery, were associated with favourable survival.

**Conclusions:**

Elective AAR demonstrates low perioperative risk and stable long-term survival, significantly influenced by annual institutional volume and clinical phenotypes. These findings support dedicated aortic centres and individualized risk stratification.

## Graphical abstract

**Figure:**
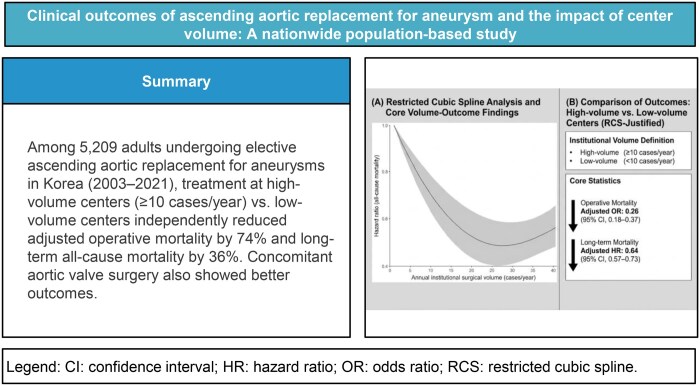

## INTRODUCTION

The 2024 European Association for Cardio-Thoracic Surgery (EACTS) and Society of Thoracic Surgeons (STS) updated guidelines lowered the elective surgery threshold for ascending thoracic aortic aneurysms (ATAA) to >50 or 52 mm, replacing the 55 mm cut-off.^1^^,^^2^ This Class IIa recommendation relies on Level of Evidence C, derived mostly from small cohorts and retrospective analyses.^3^^,^^4^ To define optimal treatment thresholds, natural history risks must be weighed against expected surgical morbidity and mortality. Given the inherent complexity of proximal aortic surgery, these surgical risks depend on institutional case volume and multidisciplinary expertise.^5^^,^^6^

While previous studies have evaluated ascending aortic replacement (AAR) outcomes and the volume-outcome relationship, most relied on single-centre or regional registries.^7^^,^^8^ Utilizing the comprehensive Korean National Health Insurance Service (NHIS) database—covering >97% of the national population—we identified all patients undergoing AAR for ATAA since 2003. We aimed to characterize patient risk profiles, provide contemporary safety benchmarks supporting evolving guidelines, and quantitatively evaluate the continuous centre volume-outcome relationship on a nationwide scale.

## METHODS

### Ethical statement

The Institutional Review Board of Seoul National University Hospital approved this study (E-2207-068-1339; consent waived). NHIS database utilization was approved and monitored in accordance with the WMA Declaration of Taipei.

### Data source and patient selection

Data were obtained from the Korean NHIS claims database.^9^^,^^10^ Because diagnostic (International Classification of Diseases, 10th Revision, Clinical Modification [ICD-10]) and procedural codes are mandatory for insurance reimbursement in South Korea, unrecorded codes indicated the “absence of disease” or “non-use” of a procedure; therefore, data imputation for missing covariates was not required.

Adult patients (≥19 years) who underwent elective AAR (code O2031) from January 2003 to December 2021 were included. Exclusions comprised acute aortic dissection, rupture, prior dissection, concomitant arch/descending aortic replacement, and emergency cases. Patients undergoing additional procedures, including full aortic root replacement or concomitant aortic valve surgery (cAVS), were included unless meeting exclusion criteria. Preoperative comorbidities were defined by diagnosis codes within 1 year before surgery, and the Charlson comorbidity index (CCI) was calculated^11^ (**Tables S1 and S2**).

### Evaluation of clinical outcomes

Operative mortality was death during the index admission. Postoperative stroke required objective neuroimaging confirmation by a specialist. Reoperation included subsequent surgical or endovascular aortic interventions. All-cause mortality was obtained from Statistics Korea. Follow-up for all long-term outcomes was closed on December 31, 2022, achieving 100% completeness.

### Statistical analysis

Continuous variables are presented as mean (SD) or median (interquartile range, IQR); categorical variables are frequencies (%). A restricted cubic spline (RCS) model evaluated the continuous exposure-response relationship between institutional volume and outcomes. The independent variable was defined as average annual procedural volume (cases/year) to prevent immortal-time bias associated with cumulative metrics. RCS provided a flexible, parsimonious approach to modelling non-linear associations, reducing overfitting risks. The model was fitted using 3 prespecified knots selected via optimal fit statistics (Akaike Information Criterion: 16174; Bayesian Information Criterion: 14188).

To identify a data-driven inflection point, we applied the stratum-specific likelihood ratio method,^12^ which calculates likelihood ratios across varying volume strata. Based on these evaluations—confirming early initiation of survival benefits—and clinical pragmatism regarding institutional expertise, a high-volume centre was formally defined as performing ≥10 cases/year.

Early and long-term outcomes were evaluated using multivariable logistic and Cox proportional hazards models, reporting odds ratios (ORs) or hazard ratios (HRs) with 95% CIs. Candidate covariates were selected *a priori*. All models met the requirement for events-per-variable (EPV > 10), with no evidence of overfitting. The proportional hazards assumption, verified via Schoenfeld residuals and log-minus-log plots, was met after categorizing age into 20-year intervals (**Figure S1**). To address time-dependent volume effects across surgical eras, a time-stratified Cox model was utilized. Kaplan-Meier survival curves were truncated when <10% of patients remained at risk (at 18 years) to avoid tail-behaviour over-interpretation. Internal validation used Hosmer-Lemeshow tests and Harrell’s *C*-index. Sensitivity analyses adjusted for surgical eras and excluded extremely low-volume centres (≤1 case/year) to test robustness. Analyses were performed using SAS Enterprise Guide 7.1 (SAS Institute, Cary, NC, United States) and R software version 4.0.3 (R Foundation for Statistical Computing, Vienna, Austria). *P* < .05 was considered significant.

## RESULTS

### Patient characteristics

**Figure 1.**
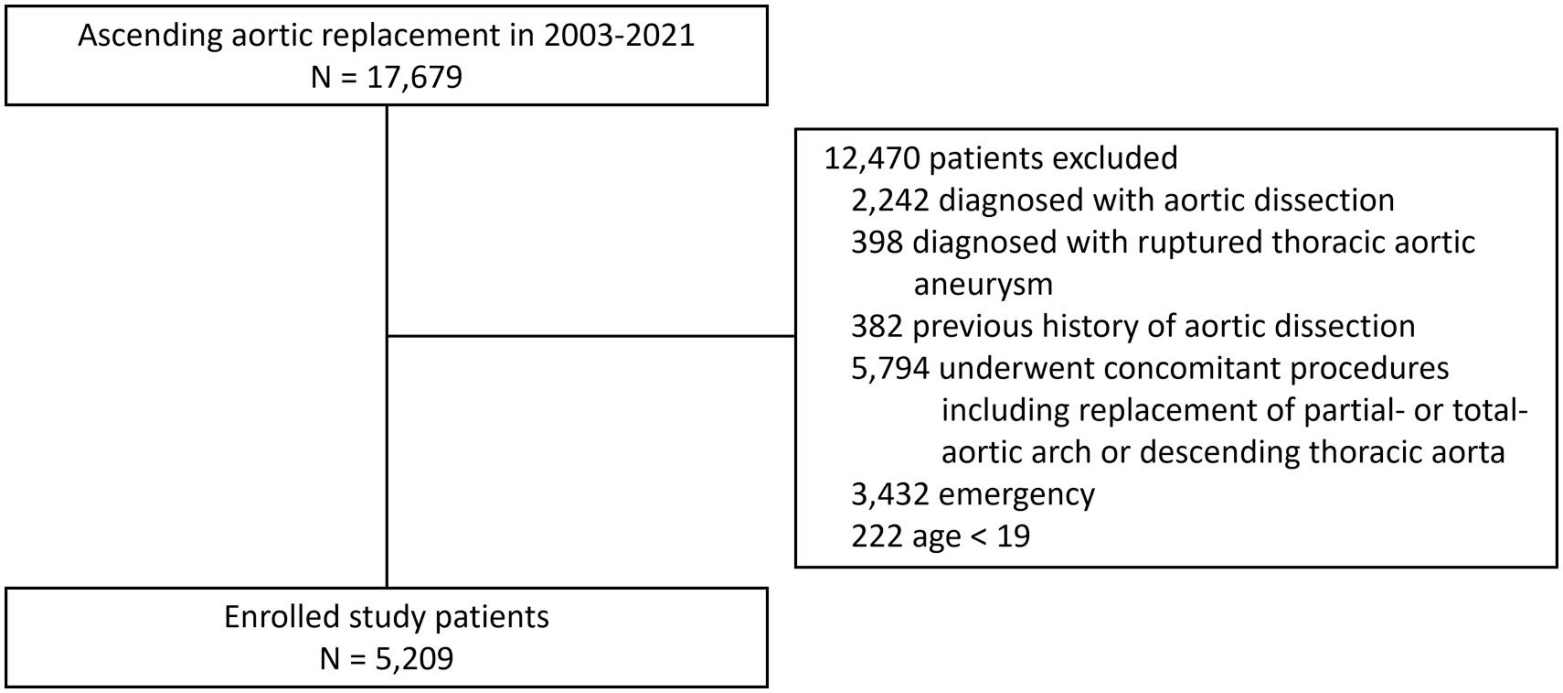
Flow Diagram of Patient Selection

Among 17 679 initially identified patients, exclusions yielded 5209 eligible patients (**Figure 1**). Median age was 62 years; 64.7% were male. Mean CCI was 2.4, and bicuspid aortic valve (BAV) prevalence was 22.3%. Concomitant procedures were performed in 85.9%, mostly cAVS (82.6%). Isolated AAR patients had more prior cardiac surgeries (31.2% vs 0.0%), cancer, and extracardiac vascular diseases than cAVS patients. Conversely, cAVS patients exhibited more cardiac-specific comorbidities and higher BAV prevalence (25.0% vs 10.3%). BAV patients were significantly younger with fewer systemic comorbidities than non-BAV patients (**Table 1**).

**Table 1.** Baseline Characteristics and Risk Factors of the Study Population

Variable	Total (*n* = 5209)	Isolated AAR (*n* = 732)	cAVS (*n* = 4300)	*P*-value	BAV (*n* = 1161)	Non-BAV (*n* = 4048)	*P*-value
Sex, male, *n* (%)	3372 (64.7)	392 (53.6)	2890 (67.2)	<.001	841 (72.4)	2526 (62.4)	<.001
Age, median (IQR)	62 (51-70)	62 (51-72)	61 (51-69)	.005	60 (51-67)	62 (51-71)	<.001
Age group, *n* (%)				.684			<.001
19-39 years	607 (11.7)	84 (11.5)	505 (11.7)		114 (9.8)	493 (12.2)	
40-59 years	1668 (32.0)	228 (31.2)	1401 (32.6)		458 (39.5)	1210 (29.9)	
≥60 years	2934 (56.3)	420 (57.4)	2394 (55.7)		589 (50.7)	2345 (57.9)	
Charlson comorbidity index, mean (SD)	2.4 (2.1)	2.5 (2.2)	2.4 (2.1)	.109	2.2 (2.0)	2.5 (2.1)	<.001
Risk factors, *n* (%)							
Diabetes mellitus	1453 (27.9)	194 (26.5)	1206 (28.1)	.389	313 (27.0)	1140 (28.2)	.421
Hypertension	3886 (74.6)	553 (75.6)	3189 (74.2)	.428	766 (66.0)	3120 (77.1)	<.001
Dyslipidaemia	2804 (53.8)	393 (53.7)	2307 (53.7)	.985	664 (57.2)	2140 (52.9)	.009
Chronic lung disease	1855 (35.6)	264 (36.1)	1522 (35.4)	.726	390 (33.6)	1465 (36.2)	.103
Cerebrovascular disease	758 (14.6)	131 (17.9)	597 (13.9)	.004	145 (12.5)	613 (15.1)	.024
Renal disease	203 (3.9)	32 (4.4)	161 (3.7)	.414	20 (1.7)	183 (4.5)	<.001
Liver disease	211 (4.1)	36 (4.9)	171 (4.0)	.236	53 (4.6)	158 (3.9)	.313
Atrial fibrillation	2124 (40.8)	87 (11.9)	526 (12.2)	.791	116 (10.0)	554 (13.7)	.001
Coronary artery disease	670 (12.9)	248 (33.9)	1794 (41.7)	<.001	443 (38.2)	1681 (41.5)	.039
Bicuspid aortic valve	1161 (22.3)	75 (10.3)	1076 (25.0)	<.001			
Congestive heart failure	2061 (39.6)	210 (28.7)	1777 (41.3)	<.001	404 (34.8)	1657 (40.9)	<.001
Peripheral vascular disease	506 (9.7)	87 (11.9)	397 (9.2)	.024	104 (9.0)	402 (9.9)	.324
Cancer	432 (8.3)	92 (12.6)	317 (7.4)	<.001	86 (7.4)	346 (8.6)	.214
Previous cardiac surgery	228 (4.4)	228 (31.2)	0 (0.0)	<.001	22 (1.9)	206 (5.1)	<.001
Concomitant procedure, *n* (%)							
Aortic valve surgery	4300 (82.6)				1076 (92.7)	3224 (79.6)	<.001
Mitral valve surgery	471 (9.0)		405 (9.4)		50 (4.3)	421 (10.4)	<.001
Tricuspid valve surgery	232 (4.5)		190 (4.4)		18 (1.6)	214 (5.3)	<.001
Arrhythmia surgery	334 (6.4)		291 (6.8)		60 (5.2)	274 (6.8)	.050
Coronary artery bypass grafting	1554 (29.8)		1465 (34.1)		234 (20.2)	1320 (32.6)	<.001

Abbreviations: AAR, ascending aortic replacement; BAV, bicuspid aortic valve; cAVS, concomitant aortic valve surgery; IQR, interquartile range.

### Overall trends in Korea

**Figure 2.**
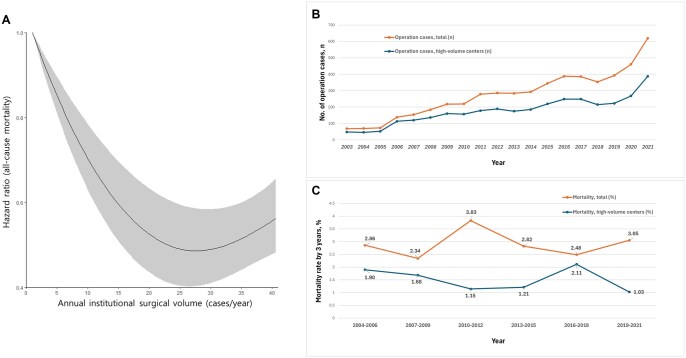
Surgical Volume Trends and Volume-Outcome Relationships. (**A**) Restricted cubic spline curve of annual institutional volume versus adjusted long-term mortality hazard. Solid line and shaded area represent the hazard ratio (HR) and 95% CIs. Reference (HR = 1.0): 1-2.5 cases/year. (**B**) Annual procedural trends: total cohort versus high-volume centres. (**C**) Operative mortality trends: total cohort versus high-volume centres

Ascending aortic replacement procedures exhibited substantial institutional centralization. Among 92 centres nationwide, only 6 (6.5%) met the high-volume criteria (≥10 cases/year), yet accounted for 64.6% of all procedures (3367/5209). The 3-knot RCS model confirmed a non-linear inverse association between annual institutional volume and long-term mortality (*P* for non-linearity <.001; **Figure 2A**). Stratum-specific likelihood ratio analysis identified that a survival benefit initiates at approximately 2.7 cases/year (HR, 0.52). The implemented threshold of ≥10 cases/year resides firmly within this statistically verified benefit zone, serving as a clinically pragmatic benchmark. Annual volumes increased consistently post-2017 (**Figure 2B**). High-volume centres consistently maintained lower operative mortality, reaching 1.03% in 2019-2021 (**Figure 2C**).

### Early clinical outcomes

Overall operative mortality and postoperative stroke rates were 2.9% and 2.2%, respectively. In high-volume centres, crude operative mortality and stroke rates were 1.4% and 1.8%. After multivariable adjustment, treatment at high-volume centres was associated with significantly lower operative mortality (adjusted OR, 0.26; 95% CI, 0.18-0.37; *P* < .001). In the isolated AAR subgroup, the unadjusted operative mortality and stroke rates were 5.3% and 3.8%, respectively.

### Long-term clinical outcomes

**Figure 3.**
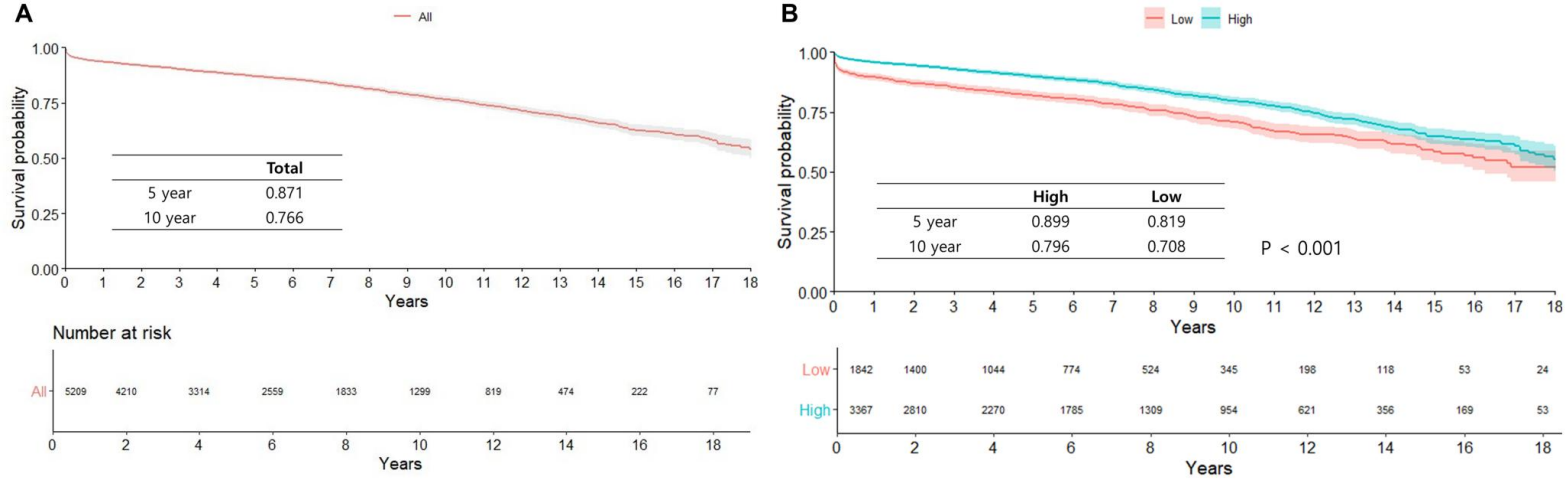
Kaplan-Meier Estimates of Long-Term Overall Survival. (**A**) Entire cohort. (**B**) Stratified by institutional volume (high-volume vs low-volume centres). Shaded areas: 95% CIs. Curves truncated at 18 years (<10% at risk) to avoid over-interpreting tail behaviour

During a 5.9-year median follow-up (IQR 2.5-9.5), 5- and 10-year survival rates were 87.1% and 76.6% (**Figure 3A**). Survival was significantly higher in high-volume centres (10-year, 79.6% vs 70.8%; **Figure 3B**), remaining robust after multivariable adjustment (adjusted HR, 0.64; 95% CI, 0.57-0.73; *P* < .001). All-cause mortality incidence was 3.0 per 100 patient-years overall, 2.5 in high-volume centres, and 4.2 in isolated AAR patients. Reoperation was required in 2.2% of patients.

### Risk factor analysis and model validation

Multivariable logistic regression (**Table 2**) identified renal disease, prior cardiac surgery, and concomitant mitral surgery as independent risk factors for operative mortality. High-volume centres (OR, 0.26), BAV (OR, 0.51), and cAVS (OR, 0.50) were associated with significantly reduced mortality risk. In the isolated AAR subgroup, high-volume centres showed lower mortality risk (OR, 0.32) (**Table S3**). For postoperative stroke (**Table S4**), concomitant mitral surgery increased risk (OR, 2.05), while cAVS was associated with a lower risk (OR, 0.49). For the isolated AAR subgroup, an adjusted multivariable model for stroke was not constructed, as limited events (*n* = 28) precluded robust analysis (**Table S5**).

**Table 2:** Multivariable Logistic Regression Analysis for Predictors of Operative Mortality

	Univariable		Multivariable	
Variables	OR (95% CI)	*P*-value	OR (95% CI)	*P*-value
Sex (male)	0.64 (0.46-0.88)	.007	0.77 (0.55-1.08)	.129
Age group				
19-39 years	1 (Reference)		1 (Reference)	
40-59 years	0.76 (0.37-1.56)	.453	0.58 (0.27-1.22)	.149
≥60 years	2.25 (1.21-4.20)	.011	1.51 (0.77-2.94)	.232
Charlson comorbidity index	1.13 (1.06-1.21)	<.001	0.91 (0.79-1.04)	.127
Surgery volume				
High-volume (≥10 cases/year)	0.25 (0.18-0.36)	<.001	0.26 (0.18-0.37)	<.001
Risk factors				
Diabetes mellitus	1.55 (1.10-2.16)	.011	1.63 (0.97-2.72)	.064
Hypertension	1.33 (0.89-1.98)	.165		
Dyslipidaemia	0.99 (0.72-1.37)	.963		
Chronic lung disease	1.23 (0.89-1.72)	.213		
Cerebrovascular disease	1.54 (1.03-2.30)	.036	1.29 (0.81-2.04)	.284
Renal disease	3.32 (1.97-5.62)	<.001	2.77 (1.47-5.22)	.002
Liver disease	1.52 (0.77-3.03)	.230		
Atrial fibrillation	1.53 (1.11-2.12)	.010	1.16 (0.75-1.81)	.508
Coronary artery disease	1.94 (1.31-2.88)	.001	1.37 (0.97-1.95)	.076
Bicuspid aortic valve	0.29 (0.16-0.53)	<.001	0.51 (0.28-0.94)	.030
Congestive heart failure	1.53 (1.10-2.11)	.011	1.31 (0.89-1.91)	.168
Peripheral vascular disease	1.27 (0.77-2.09)	.353		
Cancer	0.87 (0.47-1.61)	.649		
Previous cardiac surgery	3.56 (2.18-5.82)	<.001	2.45 (1.33-4.49)	.004
Concomitant procedure				
Aortic valve surgery	0.32 (0.23-0.45)	<.001	0.50 (0.33-0.76)	.001
Mitral valve surgery	2.37 (1.56-3.62)	<.001	2.37 (1.44-3.90)	.001
Tricuspid valve surgery	2.27 (1.29-4.00)	.005	1.60 (0.82-3.12)	.166
Arrhythmia surgery	1.64 (0.95-2.83)	.076		
Coronary artery bypass grafting	0.97 (0.68-1.38)	.851		

Abbreviation: OR, odds ratio.

Cox regression for long-term mortality (**Table 3**) demonstrated independent associations with older age, higher CCI, renal/liver disease, atrial fibrillation, prior cardiac surgery, and concomitant mitral surgery. High-volume centres (HR, 0.64), BAV (HR, 0.61), and arrhythmia surgery were associated with reduced hazard risks. These findings remained consistent within the isolated AAR subgroup (**Table S6**).

**Table 3.** Multivariable Cox Proportional Hazards Regression Analysis for Predictors of Long-Term All-Cause Mortality

	Univariable		Multivariable	
Variables	HR (95% CI)	*P*-value	HR (95% CI)	*P*-value
Sex (male)	0.91 (0.80-1.04)	.153		
Age group				
19-39 years	1 (Reference)		1 (Reference)	
40-59 years	1.78 (1.29-2.48)	.001	1.54 (1.11-2.15)	.011
≥60 years	5.23 (3.86-7.09)	<.001	4.00 (2.91-5.49)	<.001
Charlson comorbidity index	1.21 (1.18-1.24)	<.001	1.11 (1.03-1.19)	.006
Surgery volume				
High-volume (≥10 cases/year)	0.63 (0.55-0.71)	<.001	0.64 (0.57-0.73)	<.001
Risk factors				
Diabetes mellitus	1.56 (1.37-1.78)	<.001	0.89 (0.70-1.13)	.337
Hypertension	1.76 (1.50-2.07)	<.001	1.12 (0.94-1.33)	.205
Dyslipidaemia	1.10 (0.97-1.24)	.134		
Chronic lung disease	1.37 (1.21-1.55)	<.001	0.98 (0.85-1.15)	.838
Cerebrovascular disease	1.79 (1.53-2.08)	<.001	1.09 (0.91-1.30)	.358
Renal disease	3.00 (2.37-3.81)	<.001	1.74 (1.31-2.31)	<.001
Liver disease	1.55 (1.20-2.00)	.001	1.33 (1.01-1.76)	.041
Atrial fibrillation	1.98 (1.70-2.31)	<.001	1.56 (1.30-1.87)	<.001
Coronary artery disease	1.46 (1.29-1.65)	<.001	1.06 (0.93-1.20)	.417
Bicuspid aortic valve	0.49 (0.41-0.60)	<.001	0.61 (0.50-0.74)	<.001
Congestive heart failure	1.52 (1.35-1.72)	<.001	1.07 (0.92-1.24)	.373
Peripheral vascular disease	1.33 (1.09-1.63)	.005	0.84 (0.67-1.04)	.111
Cancer	1.84 (1.51-2.23)	<.001	1.13 (0.87-1.45)	.363
Previous cardiac surgery	1.87 (1.45-2.40)	<.001	1.63 (1.21-2.19)	.001
Concomitant procedure				
Aortic valve surgery	0.63 (0.55-0.73)	<.001	0.86 (0.72-1.02)	.084
Mitral valve surgery	1.46 (1.22-1.75)	<.001	1.34 (1.10-1.64)	.005
Tricuspid valve surgery	1.66 (1.30-2.11)	<.001	1.19 (0.91-1.55)	.218
Arrhythmia surgery	1.37 (1.09-1.72)	.008	0.66 (0.51-0.86)	.002
Coronary artery bypass grafting	0.80 (0.70-0.93)	.003	1.01 (0.86-1.18)	.921

Abbreviation: HR, hazard ratio.

Interaction testing revealed a significant interaction between institutional volume and recent surgical eras. A time-stratified Cox model showed high-volume centres were associated with favourable survival pre-2017 (HR, 0.69; 95% CI, 0.59-0.80; *P* < .001) and demonstrated a more pronounced association post-2017 (HR, 0.48; 95% CI, 0.37-0.64; *P* < .001).

All models met EPV requirements (>10) without overfitting. Hosmer-Lemeshow tests and calibration plots confirmed agreement between predicted and observed probabilities (Harrell’s *C*-index = 0.716; **Figure S2**).

Multiple sensitivity analyses confirmed findings. First, adjusting for discrete eras showed the recent era (2019-2021) was not significantly associated with lower operative mortality versus the early era (2003-2011) (*P* = .283). High-volume centres remained an independent survival predictor (OR, 0.26; HR, 0.64), indicating the volume effect persists regardless of secular trends (**Table S7**). Second, applying alternative thresholds of ≥15 cases/year yielded identical centre stratification due to volume distribution. A stricter threshold of ≥20 cases/year demonstrated a highly consistent independent survival benefit (adjusted HR, 0.67; 95% CI, 0.59-0.76; *P* < .001). Finally, excluding extremely low-volume centres showed consistent results (**Table S8**).

## DISCUSSION

We evaluated contemporary clinical outcomes of elective AAR across nearly 2 decades. With over 5000 cases from the Korean NHIS, the main findings are: (1) extreme centralization exists, with 6.5% of centres handling 64.6% of the volume; (2) high institutional volume (≥10 cases/year) showed a continuous inverse association with mortality; (3) isolated AAR had higher early and late risks, while cAVS was associated with favourable survival; and (4) BAV patients demonstrated superior outcomes.

The 2.9% overall mortality (1.4% in high-volume centres) is comparable to international reports.^5^^,^^13–15^ Demonstrating that elective AAR carries low operative risk nationwide provides a crucial real-world safety benchmark, strongly supporting recent EACTS/STS guidelines favouring lower surgical thresholds.^1^^,^^2^

A notable finding is the extreme centralization and established volume-outcome relationship.^6^^,^^13^^,^^16^^,^^17^ Although operative mortality declined over 2 decades—suggesting a secular trend driven by surgical advancements—our era-adjusted analysis confirmed this does not offset the volume effect. Treatment at high-volume centres independently reduced operative mortality (adjusted OR, 0.26) and long-term mortality (adjusted HR, 0.64). These results indicate “centre volume” acts as a broad surrogate encompassing unmeasured institutional competencies—dedicated aortic teams, specialized intensive care, and standardized protocols.^18^ Consequently, managing complex aortic patients at specialized centres optimizes outcomes.

While international literature^5^ utilizes volume thresholds ranging from 30 to 50 cases/year for proximal aortic procedures, our data-driven likelihood ratio analysis indicated that survival advantages initiate at a lower volume. Applying an international standard would be contextually unreflective of this cohort due to patterns of extreme centralization. Sensitivity analyses utilizing alternative cut-offs of ≥15 cases/year (yielding identical centre stratification due to data distribution) and ≥20 cases/year confirmed that the observed association with favourable long-term survival remained consistent (adjusted HR, 0.67; 95% CI, 0.59-0.76; *P* < .001), demonstrating the robustness of the volume-outcome relationship.

Unexpectedly, isolated AAR patients experienced substantially worse outcomes. This survival gap is partially driven by case-mix differences; the isolated AAR cohort was heavily enriched with patients having prior cardiac surgeries (31.2% vs 0.0%) and aggressive primary aortopathies. Conversely, the cAVS group exhibited a significantly higher baseline burden of cardiac-specific comorbidities, including congestive heart failure and coronary artery disease, as well as a high prevalence of concomitant coronary artery bypass grafting (34.1%) (**Table 1**). Crucially, even after statistically adjusting for these contrasting profiles, cAVS remained independently associated with favourable survival. This suggests the observed difference is not merely residual confounding but stems from distinct phenotypic trajectories. Favourable survival in cAVS does not imply a direct biological causal effect of valve surgery. Rather, primary valvular dysfunction likely prompts closer clinical surveillance and earlier surgical intervention.^14^ In contrast, patients requiring isolated AAR represent a more vulnerable phenotype with severe primary aortopathy, potentially involving unrecognized heritable thoracic aortic disease.^7^^,^^15^^,^^19^ Consequently, they are more susceptible to aggressive disease progression and distal aortic events during long-term follow-up.^8^^,^^20^ Therefore, relying solely on absolute diameter thresholds may inadequately capture the true risk profile of vulnerable isolated aneurysms. Future risk stratification should integrate individualized metrics like the aortic height index^21^ and biomechanical wall stress.^22^ Importantly, even within this higher-risk isolated AAR cohort, operation at high-volume centres substantially reduced long-term mortality (HR, 0.32), emphasizing the importance of referring vulnerable patients to specialized centres.

Similarly, BAV patients demonstrated markedly superior survival. This advantage stems from phenotypic differences: BAV patients carry fewer systemic comorbidities and present with primary valvular pathology prompting earlier intervention (ie, “less aortopathy”). Furthermore, BAV-associated aortopathy exhibits a biologically less aggressive natural history.^4^ Histological studies confirm that tricuspid-associated aneurysms feature pronounced degenerative changes—severe cystic medial necrosis, elastic fibre fragmentation, and extensive fibrosis—which are notably milder in BAV-associated aortas.^23–26^

### Limitations

This study has limitations. First, relying on the NHIS administrative claims database using ICD-10 codes carries a risk of miscoding. Second, granular preoperative clinical parameters, risk scores, and precise anatomical imaging data were unavailable. Third, procedural granularity and intraoperative metrics—such as aortic cross-clamp time, extracorporeal circulation time, cerebral perfusion strategies, and cannulation methods—were unavailable. Furthermore, administrative claims codes cannot reliably differentiate an extensive full aortic root replacement (Bentall procedure) from a supracoronary replacement with concomitant valve surgery. While this introduces procedural heterogeneity, we deliberately retained this comprehensive sample to preserve real-world external validity, explicitly adjusting for concomitant procedures in our multivariable models. Advanced propensity-adjusted comparisons were unfeasible, as the absence of anatomical imaging precluded balancing distinct pathophysiological phenotypes. Fourth, cause-specific death was unrecorded. Due to the very low incidence of reoperation (2.2%), a formal competing risk analysis with mortality was omitted, as it would be statistically underpowered. Finally, while mortality models were well-calibrated, the stroke risk calibration deviated slightly, warranting cautious interpretation.

## CONCLUSION

Elective AAR for ATAA demonstrates a low overall operative mortality and sustained long-term survival. These outcomes, characterized by a significant inverse association between annual institutional volume and mortality, establish population-level safety benchmarks that support the updated surgical thresholds in international guidelines. Furthermore, surgical prognosis is significantly influenced by distinct clinical phenotypes, including BAV. These findings underscore the critical role of dedicated multidisciplinary aortic centres and the necessity of individualized, phenotype-driven risk stratification.

## Supplementary Material

ivag184_Supplementary_Data

## Data Availability

The data are available on reasonable request to the corresponding author.

## ABBREVIATIONS

AAR: ascending aortic replacement
ATAA: ascending thoracic aortic aneurysm
BAV: bicuspid aortic valve
cAVS: concomitant aortic valve surgery
CCI: Charlson comorbidity index
EACTS: European Association for Cardio-Thoracic Surgery
EPV: events-per-variable
HR: hazard ratio
ICD-10: International Classification of Diseases, 10th Revision, Clinical Modification
IQR: interquartile range
NHIS: National Health Insurance Service
OR: odds ratio
RCS: restricted cubic spline
STS: Society of Thoracic Surgeons
