# Supplementary material for: Clinical Outcomes of Ascending Aortic Replacement for Aneurysm and the Impact of Centre Volume: A Nationwide Population-Based Study
Source: Interdiscip Cardiovasc Thorac Surg. 2026 Jul 27;41(8):ivag184. doi: 10.1093/icvts/ivag184 (PMC13431126; doi:10.1093/icvts/ivag184)
Supplement: ivag184_Supplementary_Data [file ivag184_supplementary_data.docx]

**Supplementary Materials**


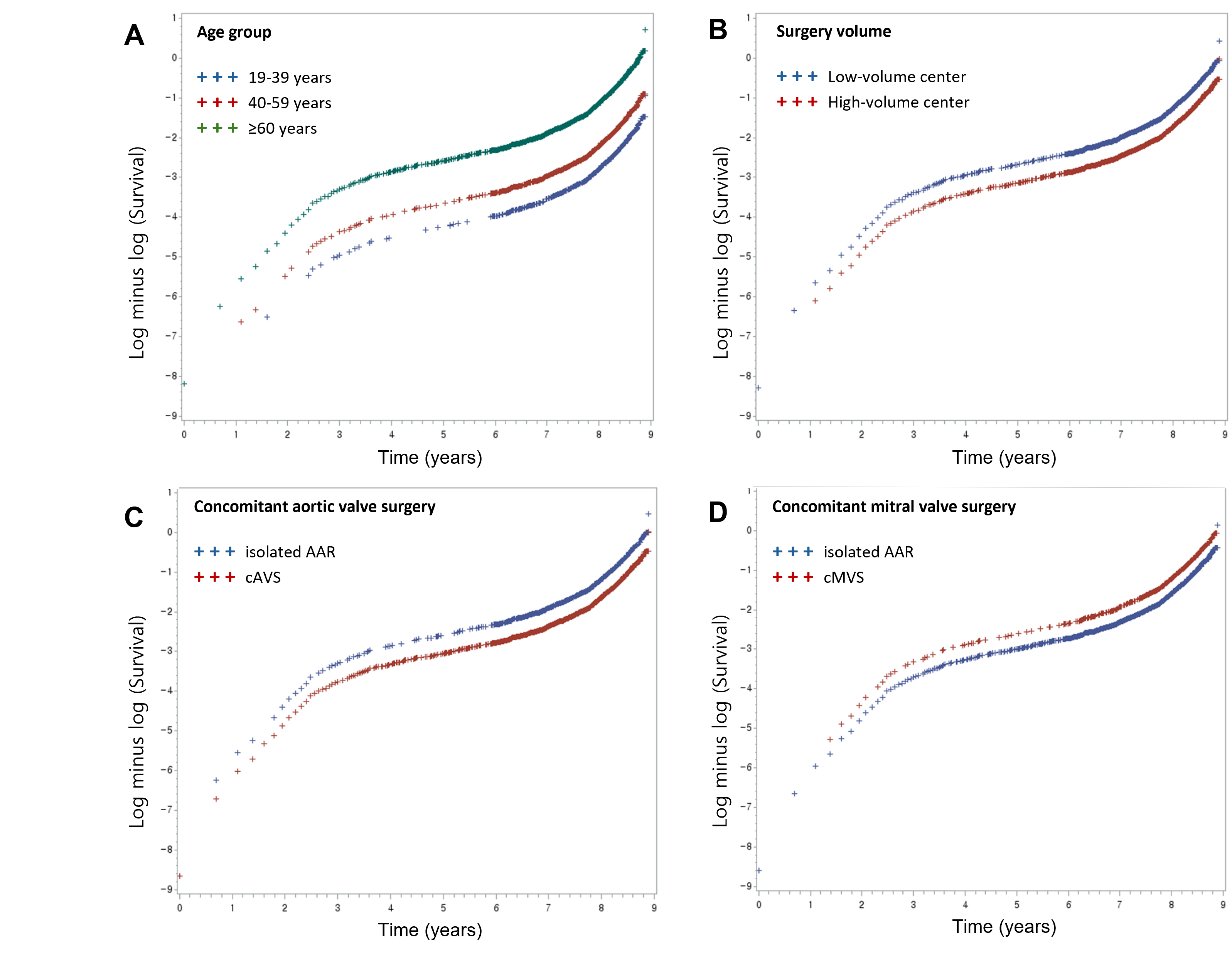
**Figure S1:** Log-minus-log survival plots assessing the proportional hazards assumption for the multivariable Cox regression model. Plots are displayed for key categorical variables: **(A)** age group, **(B)** surgery volume, **(C)** concomitant aortic valve surgery, and **(D)** concomitant mitral valve surgery.

**Figure S2:** Calibration plots of the multivariable models for **(A)** operative mortality, **(B)** postoperative stroke, and **(C)** long-term all-cause mortality.


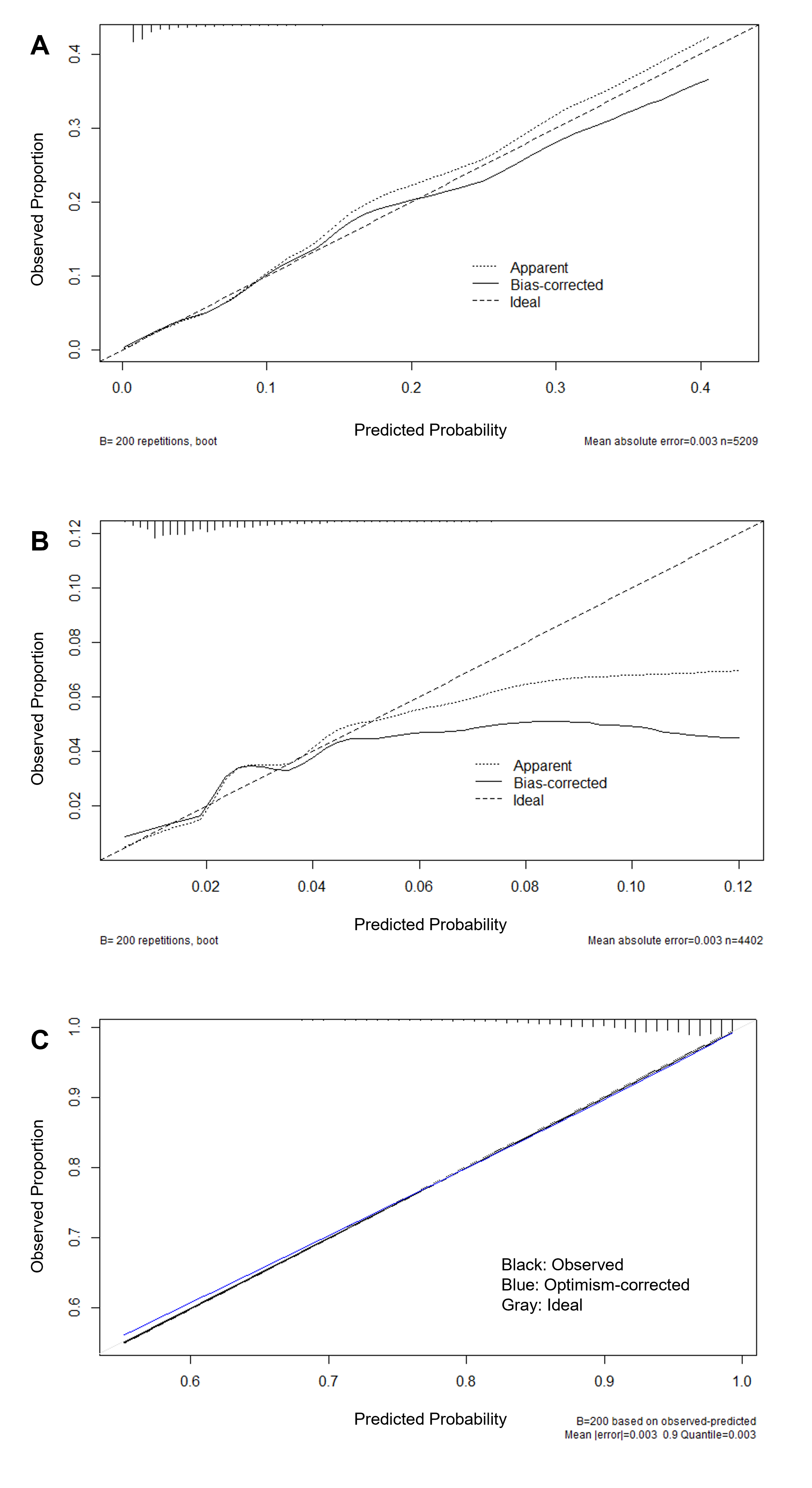
The x-axis represents the predicted probability of the event, and the y-axis represents the actual observed proportion. The diagonal dashed line indicates perfect calibration (ideal), where the predicted probability exactly matches the observed proportion. The dotted line represents the apparent performance, and the solid line represents the optimism-corrected (bias-corrected) performance of the models using bootstrapping. The tick marks at the top of the plot (rug plot) indicate the distribution of the predicted probabilities.

**Table S1:** ICD-10 diagnosis codes used for the evaluation of preoperative comorbidities.

| Preoperative comorbidities | Diagnosis codes (ICD-10) |
| --- | --- |
| Hypertension | I10.x–I13.x, I15.x |
| Diabetes mellitus | E10, E10.0, E10.1, E10.9, E11, E11.0, E11.1, E11.9, E12.0, E12.1, E12.9, E13.0, E13.1, E13.9, E14.0, E14.1, E14.9, E10.2–8, E11.2–8, E12.2–8, E13.2–8, E14.2–8 |
| Dyslipidemia | E78.0–E78.5 |
| Chronic lung disease | J40.x–J47.x, J60.x–J67.x, J68.4, J70.1, J70.3 |
| Cerebrovascular disease | G45.x, G46.x, H34.0–2, I60.x–I69.x |
| Ischemic stroke | H34.0–2, I63.x–I66.x, I69.3–I69.4 |
| Hemorrhagic stroke | I60.x–I62.x, I69.0–I69.2 |
| Renal disease | N18.x, N19, N26, Z49.0–Z49.2, Z94.0, Z99.2 |
| Liver disease | K70.2, K70.3, K71.7, K73.x, K74.x, K76.1, K72.1, K72.9, K76.6, K76.7 |
| Atrial fibrillation | I48, I480, I481, I482, I483, I484, I489 |
| Coronary artery disease | I20.x, I21.x, I22.x, I23.x, I24.x, I25.x |
| Congestive heart failure | I11.0, I13.0, I13.2, I25.5, I42.0, I42.5–I42.9, I43.x, I50.x, P29.0 |
| Peripheral vascular disease | I70.22–25, I70.29, I73.1, I73.80, I73.9, I74.0, I77.1, I79.2 |

ICD-10, International Classification of Diseases, 10th Revision, Clinical Modification.

**Table S2**: Medical conditions, ICD-10 diagnosis codes, and weighted values used for the calculation of the Charlson comorbidity index.

| Conditions | Diagnosis codes (ICD-10) | Weighted value |
| --- | --- | --- |
| Myocardial infarction | I21.x, I22.x, I23.x, I25.2 | 1 |
| Congestive heart failure | I11.0, I13.0, I13.2, I25.5, I42.0, I42.5–9, I43.x, I50.x, P29.0 | 1 |
| Peripheral vascular disease | I70.22–25, I70.29, I73.1, I73.80, I73.9, I74.0, I77.1, I79.2 | 1 |
| Cerebrovascular disease | G45.x, G46.x, H34.0–2, I60.x–I69.x | 1 |
| Dementia | B22.0, E88.8, F00.x–F03.x, F05.1, F07.2, F10.7, F13.7, F19.7, G10, G20, G30, G31.00, G31.1, G31.82 | 1 |
| Chronic pulmonary disease | J40.x–J47.x, J60.x–J67.x, J68.4, J70.1, J70.3 | 1 |
| Connective tissue disease | M05.x, M060.x, M063.x, M068.x, M069.x, M315, M32.x–M36.x | 1 |
| Ulcer disease | K25.x–K28.x | 1 |
| Mild liver disease | K70.2, K70.3, K71.7, K73.x, K74.x, K76.1 | 1 |
| Diabetes | E10, E10.0, E10.1, E10.9, E11, E11.0, E11.1, E11.9, E12.0, E12.1, E12.9, E13.0, E13.1, E13.9, E14.0, E14.1, E14.9 | 1 |
| Diabetes with end organ damage | E10.2–8, E11.2–8, E12.2–8, E13.2–8, E14.2–8 | 2 |
| Hemiplegia | G041, G114, G8001, G8002, G81.x, G82.x | 2 |
| Moderate or severe renal disease | N18.x, N19, N26, Z49.0–Z49.2, Z94.0, Z99.2 | 2 |
| Any tumor without metastasis | C00–C26, C30–C34, C37–C41, C43, C45–C58, C60–C76, C81–C85, C88, C90–C97 | 2 |
| Moderate or severe liver disease | K72.1, K72.9, K76.6, K76.7 | 3 |
| Metastatic solid tumor | C77–C80 | 3 |
| AIDS | B20, B21, B22, B23, B24 | 6 |

AIDS, Acquired Immune Deficiency Syndrome; ICD-10, International Classification of Diseases, 10th Revision, Clinical Modification.

**Table S3:** Multivariable logistic regression analysis for operative mortality in the isolated ascending aortic replacement subgroup.

|  | Univariable |  | Multivariable |  |
| --- | --- | --- | --- | --- |
| Variables | OR (95% CI) | P-value | OR (95% CI) | P-value |
| Sex (male) | 0.52 (0.27–1.02) | 0.056 |  |  |
| Age group |  |  |  |  |
| 19-39 years | 1 (Reference) |  | 1 (Reference) |  |
| 40-59 years | 0.45 (0.12–1.71) | 0.241 | 0.30 (0.07–1.22) | 0.092 |
| ≥60 years | 1.54 (0.53–4.49) | 0.430 | 1.31 (0.41–4.22) | 0.649 |
| Charlson comorbidity index | 1.17 (1.03–1.33) | 0.014 | 0.84 (0.65–1.09) | 0.192 |
| Surgery volume |  |  |  |  |
| High-volume (≥10 cases/year) | 0.41 (0.21–0.83) | 0.013 | 0.32 (0.15–0.69) | 0.003 |
| Risk factors |  |  |  |  |
| Diabetes mellitus | 2.25 (1.17–4.34) | 0.015 | 2.75 (0.99–7.63) | 0.053 |
| Hypertension | 1.83 (0.75–4.44) | 0.182 |  |  |
| Dyslipidemia | 1.12 (0.59–2.15) | 0.726 |  |  |
| Chronic lung disease | 2.41 (1.26–4.63) | 0.008 | 3.04 (1.41–6.56) | 0.005 |
| Cerebrovascular disease | 1.40 (0.65–3.03) | 0.388 |  |  |
| Renal disease | 3.63 (1.32–10.01) | 0.013 | 3.83 (1.06–13.76) | 0.040 |
| Liver disease | 1.67 (0.49–5.69) | 0.415 |  |  |
| Atrial fibrillation | 2.01 (0.89–4.52) | 0.093 |  |  |
| Coronary artery disease | 1.73 (0.90–3.30) | 0.100 |  |  |
| Bicuspid aortic valve | 0.22 (0.03–1.63) | 0.138 |  |  |
| Congestive heart failure | 2.24 (1.17–4.29) | 0.015 | 1.90 (0.89–4.04) | 0.098 |
| Peripheral vascular disease | 0.84 (0.29–2.42) | 0.747 |  |  |
| Cancer | 0.36 (0.09–1.53) | 0.167 |  |  |
| Previous cardiac surgery | 2.46 (1.28–4.70) | 0.007 | 3.05 (1.45–6.42) | 0.003 |

CI, confidence interval; OR, odds ratio**.**

**Table S4:** Multivariable logistic regression analysis for predictors of postoperative stroke.

|  | Univariable |  | Multivariable |  |
| --- | --- | --- | --- | --- |
| Variables | OR (95% CI) | P-value | OR (95% CI) | P-value |
| Sex (male) | 0.66 (0.44–0.99) | 0.045 | 0.77 (0.51–1.17) | 0.222 |
| Age group |  |  |  |  |
| 19–39 years | 1 (Reference) |  | 1 (Reference) |  |
| 40–59 years | 0.99 (0.45–2.14) | 0.972 | 0.88 (0.40–1.92) | 0.740 |
| ≥60 years | 1.87 (0.93–3.79) | 0.080 | 1.45 (0.69–3.02) | 0.328 |
| Charlson comorbidity index | 1.12 (1.02–1.23) | 0.022 | 1.03 (0.92–1.16) | 0.572 |
| Surgery volume |  |  |  |  |
| High-volume (≥10 cases/year) | 0.61 (0.41–0.92) | 0.017 | 0.67 (0.44–1.01) | 0.056 |
| Risk factors |  |  |  |  |
| Diabetes mellitus | 1.02 (0.64–1.61) | 0.949 |  |  |
| Hypertension | 1.30 (0.80–2.09) | 0.292 |  |  |
| Dyslipidemia | 1.33 (0.88–2.01) | 0.170 |  |  |
| Chronic lung disease | 1.56 (1.04–2.35) | 0.032 | 1.34 (0.86–2.09) | 0.197 |
| Cerebrovascular disease | 1.93 (1.02–3.65) | 0.045 | 1.60 (0.82–3.12) | 0.167 |
| Renal disease | 1.00 (0.31–3.19) | 0.996 |  |  |
| Liver disease | 0.79 (0.25–2.52) | 0.690 |  |  |
| Atrial fibrillation | 1.10 (0.59–2.02) | 0.772 |  |  |
| Coronary artery disease | 1.05 (0.70–1.59) | 0.805 |  |  |
| Bicuspid aortic valve | 0.61 (0.35–1.06) | 0.077 |  |  |
| Congestive heart failure | 1.03 (0.68–1.56) | 0.895 |  |  |
| Peripheral vascular disease | 1.46 (0.77–2.76) | 0.246 |  |  |
| Cancer | 1.79 (0.97–3.30) | 0.065 |  |  |
| Previous cardiac surgery | 1.66 (0.72–3.86) | 0.236 |  |  |
| Concomitant procedure |  |  |  |  |
| Aortic valve surgery | 0.45 (0.29–0.70) | <0.001 | 0.49 (0.31–0.77) | 0.002 |
| Mitral valve surgery | 1.94 (1.11–3.40) | 0.021 | 2.05 (1.16–3.61) | 0.013 |
| Tricuspid valve surgery | 0.96 (0.35–2.65) | 0.943 |  |  |
| Arrhythmia surgery | 0.87 (0.35–2.16) | 0.763 |  |  |
| Coronary artery bypass grafting | 0.71 (0.44–1.14) | 0.153 |  |  |

CI, confidence interval; OR, odds ratio**.**

**Table S5:** Univariable logistic regression analysis for postoperative stroke in the isolated ascending aortic replacement subgroup.

|  | Univariable* |  |
| --- | --- | --- |
| Variables | OR (95% CI) | P-value |
| Sex (male) | 0.82 (0.35–1.93) | 0.653 |
| Age group |  |  |
| 19-39 years | 1 (Reference) |  |
| 40-59 years | 0.22 (0.01–4.00) | 0.303 |
| ≥60 years | 2.13 (0.80–5.66) | 0.130 |
| Charlson comorbidity index | 1.16 (0.96–1.40) | 0.117 |
| Surgery volume |  |  |
| High-volume (≥10 cases/year) | 0.66 (0.28–1.57) | 0.344 |
| Risk factors |  |  |
| Diabetes mellitus | 0.92 (0.33–2.55) | 0.875 |
| Hypertension | 3.99 (0.92–17.27) | 0.192 |
| Dyslipidemia | 0.56 (0.23–1.34) | 0.523 |
| Chronic lung disease | 1.33 (0.56–3.16) | 0.160 |
| Cerebrovascular disease | 0.78 (0.10–5.96) | 0.808 |
| Renal disease | 4.45 (1.21–16.33) | 0.025 |
| Liver disease | 1.05 (0.14–8.15) | 0.962 |
| Atrial fibrillation | 0.84 (0.19–3.68) | 0.816 |
| Coronary artery disease | 1.85 (0.79–4.37) | 0.160 |
| Congestive heart failure | 1.21 (0.49–3.03) | 0.681 |
| Peripheral vascular disease | 0.93 (0.21–4.07) | 0.919 |
| Cancer | 2.29 (0.82–6.43) | 0.064 |
| Previous cardiac surgery | 0.88 (0.34–2.28) | 0.787 |

CI, confidence interval; OR, odds ratio**.** * Multivariable analysis was not performed because an adjusted analysis was not statistically feasible due to the limited number of outcome events (n=28).

**Table S6:** Multivariable Cox proportional hazards regression analysis for long-term all-cause mortality in the isolated ascending aortic replacement subgroup.

|  | Univariable |  | Multivariable |  |
| --- | --- | --- | --- | --- |
| Variables | HR (95% CI) | P-value | HR (95% CI) | P-value |
| Sex (male) | 0.95 (0.71–1.27) | 0.710 |  |  |
| Age group |  |  |  |  |
| 19-39 years | 1 (Reference) |  | 1 (Reference) |  |
| 40-59 years | 1.46 (0.76–2.81) | 0.26 | 1.47 (0.75–2.89) | 0.260 |
| ≥60 years | 3.39 (1.86–6.15) | <0.001 | 2.98 (1.54–5.76) | 0.001 |
| Charlson comorbidity index | 1.17 (1.10–1.24) | <0.001 | 1.03 (0.90–1.18) | 0.699 |
| Surgery volume |  |  |  |  |
| High-volume (≥10 cases/year) | 0.84 (0.63–1.13) | 0.247 | 0.32 (0.15–0.69) | 0.003 |
| Risk factors |  |  |  |  |
| Diabetes mellitus | 1.18 (0.85–1.63) | 0.320 | 0.77 (0.46–1.29) | 0.315 |
| Hypertension | 1.66 (1.14–2.42) | 0.008 | 0.93 (0.62–1.41) | 0.742 |
| Dyslipidemia | 0.95 (0.71–1.28) | 0.753 |  |  |
| Chronic lung disease | 1.22 (0.91–1.64) | 0.189 |  |  |
| Cerebrovascular disease | 2.12 (1.54–2.92) | <0.001 | 1.54 (1.04–2.26) | 0.029 |
| Renal disease | 3.55 (2.11–5.98) | <0.001 | 2.43 (1.36–4.32) | 0.003 |
| Liver disease | 1.63 (0.96–2.79) | 0.073 | 1.87 (1.03–3.38) | 0.038 |
| Atrial fibrillation | 1.62 (1.09–2.42) | 0.017 | 1.11 (0.71–1.73) | 0.642 |
| Coronary artery disease | 1.40 (1.04–1.88) | 0.025 | 1.07 (0.78–1.47) | 0.685 |
| Bicuspid aortic valve | 0.18 (0.07–0.48) | 0.001 | 0.24 (0.09–0.66) | 0.006 |
| Congestive heart failure | 1.65 (1.21–2.25) | 0.002 | 1.42 (0.97–2.10) | 0.070 |
| Peripheral vascular disease | 1.08 (0.68–1.71) | 0.762 |  |  |
| Cancer | 1.71 (1.14–2.57) | 0.010 | 1.41 (0.84–2.37) | 0.199 |
| Previous cardiac surgery | 1.42 (1.04–1.92) | 0.025 | 1.53 (1.08–2.16) | 0.016 |

CI, confidence interval; HR, hazard ratio**.**

**Table S7:** Sensitivity analysis—Multivariable regression models for early and long-term outcomes adjusted for surgical eras (period effects).

| **Variables** | **Operative Mortality**  **OR (95% CI)** | **P-value** | **Postoperative Stroke**  **OR (95% CI)** | **P-value** | **All-cause Mortality**  **HR (95% CI)** | **P-value** |
| --- | --- | --- | --- | --- | --- | --- |
| Sex (male) | 0.77 (0.55–1.09) | 0.141 | 0.78 (0.51–1.18) | 0.238 |  |  |
| Age (years) |  |  |  |  |  |  |
| 19–39 | 1 (Reference) |  | 1 (Reference) |  | 1 (Reference) |  |
| 40–59 | 0.58 (0.28–1.23) | 0.157 | 0.89 (0.40–1.95) | 0.767 | 1.55 (1.11–2.16) | 0.010 |
| ≥60 | 1.56 (0.80–3.07) | 0.194 | 1.48 (0.71–3.10) | 0.299 | 4.05 (2.95–5.56) | <0.001 |
| Charlson comorbidity index | 0.91 (0.79–1.04) | 0.156 | 1.04 (0.93–1.17) | 0.500 | 1.10 (1.03–1.19) | 0.008 |
| Surgery volume |  |  |  |  |  |  |
| High-volume (≥10 cases/year) | 0.26 (0.18–0.37) | <0.001 | 0.65 (0.43–0.98) | 0.042 | 0.64 (0.56–0.73) | <0.001 |
| Risk factors |  |  |  |  |  |  |
| Diabetes mellitus | 1.65 (0.99–2.78) | 0.057 |  |  | 0.91 (0.71–1.15) | 0.413 |
| Hypertension |  |  |  |  | 1.12 (0.94–1.32) | 0.209 |
| Dyslipidemia |  |  |  |  |  |  |
| Chronic lung disease |  |  | 1.32 (0.84–2.07) | 0.222 | 0.98 (0.84–1.14) | 0.801 |
| Cerebrovascular disease | 1.29 (0.81–2.05) | 0.278 | 1.61 (0.82–3.13) | 0.164 | 1.09 (0.91–1.31) | 0.336 |
| Renal disease | 2.88 (1.52–5.45) | 0.001 |  |  | 1.78 (1.34–2.37) | <0.001 |
| Liver disease |  |  |  |  | 1.34 (1.02–1.77) | 0.036 |
| Atrial fibrillation | 1.17 (0.75–1.82) | 0.486 |  |  | 1.56 (1.30–1.87) | <0.001 |
| Coronary artery disease | 1.34 (0.94–1.91) | 0.103 |  |  | 1.04 (0.92–1.19) | 0.506 |
| Bicuspid aortic valve | 0.53 (0.28–0.97) | 0.040 |  |  | 0.62 (0.51–0.76) | <0.001 |
| Congestive heart failure | 1.33 (0.91–1.95) | 0.141 |  |  | 1.08 (0.93–1.26) | 0.307 |
| Peripheral vascular disease |  |  |  |  | 0.85 (0.68–1.06) | 0.141 |
| Cancer |  |  |  |  | 1.15 (0.89–1.48) | 0.282 |
| Previous cardiac surgery | 2.45 (1.33–4.50) | 0.004 |  |  | 1.64 (1.21–2.21) | 0.001 |
| Concomitant procedure |  |  |  |  |  |  |
| Aortic valve surgery | 0.50 (0.33–0.75) | <0.001 | 0.49 (0.31–0.77) | 0.002 | 0.85 (0.71–1.01) | 0.070 |
| Mitral valve surgery | 2.35 (1.43–3.87) | <0.001 | 1.99 (1.13–3.56) | 0.018 | 1.33 (1.09–1.63) | 0.005 |
| Tricuspid valve surgery | 1.57 (0.81–3.06) | 0.185 |  |  | 1.18 (0.90–1.55) | 0.223 |
| Arrhythmia surgery |  |  |  |  | 0.66 (0.51–0.87) | 0.002 |
| CABG |  |  |  |  | 1.03 (0.88–1.20) | 0.715 |
| Surgical era |  |  |  |  |  |  |
| 2003–2011 | 1 (Reference) |  | 1 (Reference) |  | 1 (Reference) |  |
| 2012–2018 | 0.81 (0.53–1.23) | 0.324 | 0.72 (0.44–1.16) | 0.177 | 0.97 (0.83–1.13) | 0.699 |
| 2019–2021 | 0.78 (0.49–1.24) | 0.283 | 0.77 (0.45–1.33) | 0.348 | 0.80 (0.64–1.02) | 0.069 |

CABG, coronary artery bypass grafting; CI, confidence interval; HR, hazard ratio**.**

**Table S8:** Sensitivity analysis—Multivariable regression models for early and long-term outcomes after excluding extremely low-volume centers (≤1 case/year).

| **Variables** | **Operative Mortality**  **OR (95% CI)** | **P-value** | **Postoperative Stroke**  **OR (95% CI)** | **P-value** | **All-cause Mortality**  **HR (95% CI)** | **P-value** |
| --- | --- | --- | --- | --- | --- | --- |
| Sex (male) | 0.64 (0.43–0.95) | 0.026 | 0.69 (0.44–1.08) | 0.106 |  |  |
| Age (years) |  |  |  |  |  |  |
| 19–39 | 1 (Reference) |  | 1 (Reference) |  | 1 (Reference) |  |
| 40–59 | 0.60 (0.26–1.38) | 0.230 | 0.70 (0.31–1.60) | 0.399 | 1.53 (1.08–2.17) | 0.018 |
| ≥60 | 1.40 (0.67–2.97) | 0.373 | 1.37 (0.65–2.89) | 0.411 | 3.96 (2.84–5.53) | <0.001 |
| Charlson comorbidity index | 0.94 (0.82–1.09) | 0.440 | 1.06 (0.94–1.19) | 0.361 | 1.10 (1.02–1.19) | 0.011 |
| Surgery volume |  |  |  |  |  |  |
| High-volume (≥10 cases/year) | 0.36 (0.24–0.53) | <0.001 | 0.74 (0.47–1.17) | 0.203 | 0.73 (0.63–0.84) | <0.001 |
| Risk factors |  |  |  |  |  |  |
| Diabetes mellitus | 1.59 (0.88–2.86) | 0.121 |  |  | 0.93 (0.72–1.20) | 0.591 |
| Hypertension |  |  |  |  | 1.10 (0.92–1.32) | 0.296 |
| Dyslipidemia |  |  |  |  |  |  |
| Chronic lung disease |  |  | 1.34 (0.83–2.16) | 0.229 | 0.95 (0.81–1.12) | 0.556 |
| Cerebrovascular disease |  |  |  |  | 1.13 (0.93–1.37) | 0.207 |
| Renal disease | 1.99 (0.91–4.31) | 0.083 |  |  | 1.67 (1.23–2.28) | 0.001 |
| Liver disease |  |  |  |  | 1.22 (0.90–1.65) | 0.200 |
| Atrial fibrillation | 1.17 (0.75–1.82) | 0.486 |  |  | 1.56 (1.30–1.87) | <0.001 |
| Coronary artery disease | 1.40 (0.93–2.11) | 0.112 |  |  | 1.02 (0.89–1.17) | 0.799 |
| Bicuspid aortic valve | 0.49 (0.24–0.99) | 0.049 |  |  | 0.61 (0.49–0.74) | <0.001 |
| Congestive heart failure | 1.37 (0.88–2.13) | 0.162 |  |  | 1.11 (0.95–1.30) | 0.207 |
| Peripheral vascular disease |  |  |  |  | 0.81 (0.63–1.02) | 0.077 |
| Cancer |  |  |  |  | 1.19 (0.91–1.55) | 0.197 |
| Previous cardiac surgery | 3.30 (1.59–6.86) | 0.001 |  |  |  |  |
| Concomitant procedure |  |  |  |  |  |  |
| Aortic valve surgery | 0.64 (0.38–1.07) | 0.088 | 0.54 (0.33–0.89) | 0.015 | 0.82 (0.69–0.97) | 0.019 |
| Mitral valve surgery | 2.67 (1.52–4.68) | <0.001 | 2.37 (1.33–4.21) | 0.003 | 1.32 (1.07–1.64) | 0.011 |
| Tricuspid valve surgery | 1.68 (0.82–3.43) | 0.153 |  |  | 1.16 (0.88–1.54) | 0.290 |
| Arrhythmia surgery | 1.18 (0.57–2.45) | 0.659 |  |  | 0.64 (0.48–0.84) | 0.001 |
| CABG |  |  |  |  | 0.93 (0.79–1.10) | 0.411 |

CABG, coronary artery bypass grafting; CI, confidence interval; HR, hazard ratio**.**
